# VASARI 2.0: a new updated MRI VASARI lexicon to predict grading and *IDH* status in brain glioma

**DOI:** 10.3389/fonc.2024.1449982

**Published:** 2024-12-23

**Authors:** Alberto Negro, Laura Gemini, Mario Tortora, Gianvito Pace, Raffaele Iaccarino, Mario Marchese, Andrea Elefante, Fabio Tortora, Vincenzo D'Agostino, members of ODM Multidisciplinary Neuro-Oncology Group

**Affiliations:** ^1^ NeuroRadiology Unit, Ospedale del Mare, Azienda Sanitaria Locale Napoli 1 Centro (ASL NA1 Centro), Naples, Italy; ^2^ Department of Advanced Biomedical Sciences, University of Naples Federico II, Naples, Italy; ^3^ Department of Health Medicine and Science “Vincenzo Tiberio”, University of Molise, Campobasso, Italy

**Keywords:** VASARI, MRI, glioma, *IDH* status, grade tumor, neuroradiology

## Abstract

**Introduction:**

Precision medicine refers to managing brain tumors according to each patient’s unique characteristics when it was realized that patients with the same type of tumor differ greatly in terms of survival, responsiveness to treatment, and toxicity of medication. Precision diagnostics can now be advanced through the establishment of imaging biomarkers, which necessitates quantitative image acquisition and processing. The VASARI (Visually AcceSAble Rembrandt Images) manual annotation methodology is an ideal and suitable way to determine the accurate association between genotype and imaging phenotype. Our work proposes an updated version of the VASARI score that is derived by changing the evaluation ranges of its components in an effort to increase the diagnostic accuracy of the VASARI manual annotation system and to find neuroimaging biomarkers in neuro-oncology with increasing reliability.

**Materials and methods:**

We gathered the histological grade and molecular status of 126 patients with glioma (Men/Women = 75/51; mean age: 55.30) by a retrospective analysis. Two residents and three neuroradiologists blindedly examined each patient using all 25 VASARI characteristics, after having appropriately modified the reference ranges in order to implement an innovative VASARI lexicon (VASARI 2.0). It was determined how well the observers agreed. A box plot and a bar plot were used in a statistical analysis to assess the distribution of the observations. After that, we ran a Wald test and univariate and multivariate logistic regressions. To find cutoff values that are predictive of a diagnosis, we also computed the odds ratios, confidence intervals, and evaluation matrices using receiver operating characteristic curves for each variable. Finally, we performed a Pearson correlation test to evaluate whether the variable grades and *IDH* were correlated.

**Results:**

An excellent Intraclass Correlation Coefficient (ICC) estimate was obtained. In this study, five features were part of the predictive model for determining glioma grade: F4, enhancement quality [area under the curve (AUC): 0.87]; F5, tumor-enhancing proportion (AUC: 0.70); F6, tumor–non-enhancing proportion (AUC: 0.89); F7, necrosis proportion (AUC: 0.79); and F17, diffusion characteristics (AUC: 0.75). Furthermore, six features were found to predict *IDH* mutation status: F4, enhancement quality (AUC: 0.904); F5, tumor-enhancing proportion (AUC: 0.73); F6, tumor–non-enhancing proportion (AUC: 0.91); F7, necrosis proportion (AUC: 0.84); F14, proportion of edema (AUC: 0.75); and diffusion characteristics F17 (AUC: 0.79). VASARI 2.0 models showed good performances according to the AUC values, which are also compared with traditional VASARI scores.

**Discussion and conclusion:**

Glioma grade and isocitrate dehydrogenase (*IDH*) status can be predicted using specific magnetic resonance imaging (MRI) features, which have significant prognostic consequences. The accuracy of texture-derived metrics from preoperative MRI gliomas and machine learning analysis for predicting grade, *IDH* status, and their correlation can be enhanced by the suggested new and updated VASARI manual annotation system. To help with therapy selection and enhance patient care, we intend to create prediction models that incorporate these MRI findings with additional clinical data.

## 1 Introduction

The cerebral glioma, a sizable and heterogeneous family of brain tumors with various features, is the most prevalent primary malignant brain tumor that exhibits variable treatment response and patient prognosis (1). Given the considerable variations in the care of these many glioma subtypes, an accurate diagnosis is essential. Glioblastoma and oligodendroglioma, for instance, respond very differently to treatment. Furthermore, tumors belonging to the same histologic subtype could exhibit distinct behaviors in other patient cohorts. Previously, the phenotypic characteristics of the cells were used to identify the tumor grade, but, today, this seems too simplistic (2), and it is not possible to identify these distinctions between the different glioma subtypes based on the purely histology-driven older classification system. Part of the challenge was that many gliomas can contain mixed cell types, which result in high inter-observer variability of diagnosis among neuropathologists (3).

Thus, a paradigm change in the diagnosis and categorization of gliomas has resulted from new discoveries on their genetic composition.

The updated glioma classification system incorporates molecular markers into tumor subgrouping, which has been shown to better correlate with tumor biology and behavior as well as patient prognosis than the previous purely histology-based classification system (4). The isocitrate dehydrogenase (*IDH*) gene changes and the co-deletion of chromosomal arms 1 and 19 (1p/19q) are the two main alterations taken into account when differentiating tumors in the World Health Organization (WHO) classification (5). It could be comparable to biomarkers that influence the prognosis and biological behavior of a patient. For instance, it has been demonstrated that *IDH* gene family mutations offer higher overall survival in high-grade gliomas than their *IDH*–wild-type counterparts, regardless of the histological grade (6–8). Additionally, the degree of cellular differentiation and the molecular state have an impact on the course of treatment. For instance, low-grade gliomas are often not treated with adjuvant radiotherapy and/or chemotherapy. Clinicians found that patients’ responses to treatment, the severity of side effects, and even prognosis could differ even when they shared the same tumor. This implies that therapeutic care tailored to the needs of particular people or “precision medicine” may be the direction of the future (9–11). The method most frequently used today for identifying glioma mutations is immunohistochemical analysis after biopsy or surgical resection (12). By categorizing radiological gliomas in a non-invasive way with relevant prognostic consequences, clinical therapeutic planning may be recommended (13). Magnetic resonance imaging (MRI) is used as a gold standard for radiological examination of gliomas. Because there are no objective measurements that can be extensively duplicated and validated, determining the tumor grade accurately is far from simple (14).

Whereas advanced MRI techniques (e.g., diffusion Magnetic Resonance Imaging (dMRI), Perfusion Magnetic Resonance Imaging (pMRI), and Magnetic Resonance Spectroscopy (MRS)) are more specific to biophysical, cellular, and microstructural processes, conventional MRI methods (e.g., T1-weighted and T2-weighed sequences) give macrostructural anatomical evidence. Unlike standard MRI techniques, which only yield relative image contrasts, these advanced techniques have the potential to be (semi)quantitative. For the purpose of acquiring imaging biomarkers, sensitivity, specificity, and quantification are crucial (15). While a number of recent investigations have concentrated on applying sophisticated MRI methods (such as perfusion, spectroscopy, and machine learning approaches) for radiogenomic purposes (16–18), standard MRI sequences continue to be the gold standard for the investigation and characterization of brain tumors.

An optimal and adequate method to identify the right correlation between imaging phenotype and genotype, based on the evaluation of specific radiological characteristics, mainly conventional MRI features, and, at the same time, to standardize the assessment of gliomas is represented by the VASARI manual annotation system.

A collection of standardized descriptors called VASARI (Visually AcceSAble Rembrandt Images) MRI characteristics is used to describe brain tumors on contrast-enhanced MRI imaging. These characteristics aid in the diagnosis, grading, and prognostication of gliomas by offering qualitative and quantitative information regarding the visual appearance and properties of the tumor (19). The location, shape, enhancement quality, necrosis proportion, edema proportion, and other geometric parameters of the tumor are all included in the VASARI features (20).

Since its development in 2016, VASARI score has undergone a development from the number of the features, now 25, to the field of application in neuro-oncology.

Certain specific MRI features [enhancement quality (F4), tumor-enhancing proportion (F5), tumor–non-enhancing proportion (F6), and necrosis proportion (F7)] have been shown in our previously published study (21) to be predictive of the grade and *IDH* status of gliomas, with significant prognostic implications.

Inter-observer agreement and multicenter collaborations are made possible by the reliability and consistency in the interpretation of MRI scans made possible by the standardization of the VASARI features (22, 23). The communication between radiologists, oncologists, and other medical professionals involved in the treatment of patients with glioma is improved when VASARI elements are used in structured reporting systems (24). To increase the precision of glioma grading, prognosis prediction, and treatment response assessment, they have been used in machine learning algorithms and radiomics studies (24, 25). Predictive models to inform therapy choices and patient care can be created by merging VASARI variables with additional imaging features and clinical data (26, 27).

Such a model’s potential resides in its capacity to evaluate tumor features objectively. In actuality, although VASARI started out as a visual assessment scale, a numerical estimate of the features under consideration can be obtained by using “regions of interest” (ROIs) to calculate the areas of the various tumor components. However, we think that such a system is not useful in terms of outpoint prediction and is too sophisticated and challenging for doctors to utilize, especially in light of the outcomes documented in the literature and our earlier study.

In this regard, we propose a new VASARI glioma score, which we refer to as VASARI 2.0. This system evaluates only those tumor features that can be objectively described by ROI (manual segmentation) and that predict the outpoint (*IDH* status and grade) with area under the curve (AUC) > 0.8. For this purpose, we modify the evaluation intervals/ranges as explained in the following (**Table 1**), all in order to provide the scientific community with a system suitable with clinical practice.

**Table 1:** Modified ranges between VASARI and VASARI 2.0.

	*F4* *Enhancement* *quality*	*F5* *Enhanced area*	*F6* *No-enh area*	*F7* *Necrosis area*	*F14* *Edema area*	*F17* *Diffusion quality*
VASARI 2.0	1. Absent	1. Absent or <5%	1. Absent or <5%	1. Absent or <5%	1. Absent or <5%	1. Augmented
	2. Minimal	2. 6–25	2. 6–25	2. 6–25	2. 6–25	2. Reduced
	3. Avid	3. 26–50	3. 26–50	3. 26–50	3. 26–50	3. Mixed
		4. 51–75	4. 51–75	4. 51–75	4. 51–75	
		5. 76–95	5. 76–95	5. 76–95	5. 76–95	
		6. >95%	6. >95%	6. >95%	6. >95%	
VASARI	1. Absent	3. <5%	3. <5%	2. No	2. No	2. Augmented
	2. Minimal	4. 6%–33%	4. 6%–33%	3. <5%	3. <5%	3. Reduced
	3. Avid	5. 34%–67%	5. 34%–67%	4. 6%–33%	4. 6%–33%	4. Mixed
		6. 68%–95%	6. 68%–95%	5. 34%–67%	5. 34%–67%	
			7. 96%–99%			
			8. 100%			

## 2 Materials and methods

### 2.1 Ethics statement

The Institutional Review Board accepted the study because the surgery was routinely carried out and was not considered experimental. Each patient completed and signed a proper written informed consent. There was no indication of a conflict of interest from the writers. No funding was given to support this study.

### 2.2 Patient population

Retrospective analysis of patients who underwent MRI for pre-surgical glioma evaluation between 2018 and 2021 has been done on the database at our institution. Additionally, pathology reports were gathered to determine the glioma grade. According to the following criteria, a number of patients were disqualified from the study: (a) poor acquisition quality imaging; (b) no intravenous contrast; (c) medications prior to the MR examination, such as steroid medication that may alter edema and contrast enhancement; and (d) lack of a pathology report. In the end, 126 individuals with glioma were included. The study group included 51 women and 75 men, ages 14 to 84 (further information in **Table 2**).

**Table 2:** Demographic data about our study population.

		Glioma grade				
Demographic data		1 (n = 3)	2 (n = 21)	3 (n = 18)	4 (n = 84)	Total (126)
*Age (yr.)*	*< 50*	*3*	*10*	*8*	*18*	*39*
	*> 50*	*0*	*11*	*10*	*66*	*87*
*Sex*	*Male*	*2*	*8*	*11*	*54*	*75*
	*Female*	*1*	*13*	*7*	*30*	*51*
*Location*	*Frontal*	*0*	*12*	*9*	*28*	*49*
	*temporal*	*0*	*7*	*4*	*17*	*28*
	*Insular*	*2*	*2*	*1*	*6*	*11*
	*Parietal*	*0*	*0*	*1*	*22*	*23*
	*Occipital*	*0*	*0*	*2*	*2*	*4*
	*Brain steam*	*1*	*0*	*1*	*5*	*7*
	*Other (cerebellum)*	*0*	*0*	*0*	*4*	*4*
*Side*	*Right*	*0*	*11*	*5*	*47*	*63*
	*Left*	*2*	*0*	*2*	*5*	*9*
	*Central/Bilateral*	*1*	*10*	*11*	*32*	*54*
*Eloquent area*	*No*	*2*	*15*	*13*	*45*	*75*
	*Motor speech*	*1*	*2*	*1*	*7*	*11*
	*Receptive speech*	*0*	*4*	*2*	*16*	*22*
	*Motor area*	*0*	*0*	*1*	*15*	*16*
	*Visual area*	*0*	*0*	*1*	*1*	*2*
*IDH status*	*Positive*	*2*	*13*	*3*	*4*	*22*
	*Negative*	*1*	*8*	*15*	*80*	*104*

### 2.3 Image acquisition

MRI at 1.5 T (Magnetom Amyra; Siemens Medical Systems, Erlangen, Germany) was used for the imaging. In addition to T2-weighted images with dark fluid on the axial planes, the MR technique also includes T1-weighted images taken before and after the administration of gadolinium-based contrast media. In addition to this, we also carried out Diffusion Weighted Imaging (DWI) and Susceptibility Weighted Imaging (SWI) on the axial plane, as well as T1-w and T2-w sequences on additional planes. These were the precise imaging parameters: (1) axial T1-weighted MR: repetition time of 250 ms, echo time of 2.46 ms, slice thickness of 5 mm, matrix dimensions of 320 × 256, and field of view of 220 × 220 mm^2^; (2) axial T2-weighted MR: repetition time of 6000 ms, echo time of 93 ms, slice thickness of 5 mm, matrix dimensions of 320 × 288, and field of view of 198 × 220 mm; and (3) axial T2WI dark-fluid MR: repetition time of 8000 ms, echo time of 97 ms, slice thickness of 5 mm, matrix dimensions of 320 × 224, and field of view of 181 × 220 mm.

### 2.4 Magnetic resonance imaging assessment and analysis

The VASARI lexicon can be easily understood by following the specific guide downloadable from the public website of The Cancer Imaging Archive, in the specific section “Supporting Documentation and Metadata” (https://wiki.cancerimagingarchive.net/display/Public/VASARI+Research+Project).

We considered that the entire lesion was made up of necrotic tissue, edema, enhancing area, and non-enhancing area in accordance with the VASARI approach. Furthermore, we extracted the score system and morphological features. Therefore, an enhancing area was defined as any region of the tumor that shows a discernible increase in signals on the post-contrast T1-weighted pictures in comparison to those in the pre-contrast. Any region displaying T2-weighted hyperintensity (less than the intensity of the Cerebral Spinal Fluid (CSF) fluid) and corresponding T1-weighted hypointensity, as well as a mass effect and architectural distortion, such as blurring of the gray-white interface, was deemed to be a non-enhancing area. An irregular border, a high signal on T2-weighted and proton density imaging, and either no enhancement at all or a significantly decreased enhancement are characteristics of a necrotic section of the tumor. By calculating the ratio of the total lesion area to the necrosis area (internal to it), a quantitative evaluation of the necrosis was produced. On the T2-, T1-, and SWI T2*–weighted sequences, a bleeding was detected and assessed in connection with the existence of hemoglobin breakdown products. Based on an apparent diffusion coefficient (ADC) map, the diffusion characteristics are classified as mostly facilitated or restricted in the enhancing or non-contrast–enhanced tumor (nCET) region of the tumor. They are described as mixed when there is a roughly equal amount of both limited and assisted diffusion.

Three neuroradiologists, two residents and one senior, independently evaluated the imaging characteristics.

### 2.5 Statistical analysis

The aims of the statistical analysis were as follows: 1) analyze the statistically significance of each variable with respect to the prediction of the variable levels; 2) analyze the statistically significance of each variable with respect to the prediction of the variable *IDH*; and 3) analyze the relationship between *IDH* and GRADE.

To consider only relevant columns in the dataset, a sub-dataset was created, only with the following columns: GRADE, F4, F5, F6, F7, F14, F15, and F24, and another column was added to the dataset named “levels.” This variable is binary, with level = 0 denoting a grade of 1 or 2 (low grade) and level = 1 denoting a grade of 3 or 4 (high grade). The variable “*IDH* mutate” has been turned into a dummy binary variable too (*IDH* 0 = non-mutate = neg; *IDH* 1 = mutate = pos).

To see whether the variable GRADE and *IDH* are correlated, we built a contingency table where, on one side, there are the levels of GRADE and, on the other, the *IDH*, negative or positive. Then, we also did the Pearson correlation test and built a correlation plot.

As is often the case in real datasets, the VASARI dataset that we analyzed is highly imbalanced (80% vs. 20%) and is of high grade, so we divided the dataset into train (70%) and test (30%), and, then, we balanced the training data in such a way that we obtained 2,000 observation and perfectly balanced classes (p = 0.5). Then, we proceeded to the analysis of the statistically significant variables in the prediction of levels. As the first step in this part of the analysis, we conducted a multiple logistic regression using the dichotomic variable levels as response variable and each variable as covariate on the balanced training set. We also conducted a Wald test on the categorical variables to confirm statistical significance.

We computed the odds ratios (OR) and confidence intervals for each variable and evaluated the matrices with ROC curves.

After the balancing of the classing, we finally conducted a multiple logistic regression using all the variables in the dataset to have an idea of which variable is statistically significant and to see how does our classifier performs having the whole set of information in the prediction of *IDH*. We then applied this model to the test set to evaluate the predictive performance in the analysis of the relationship between *IDH* and GRADE.

We compared the results with the same value obtained in our previous studies using the gold standard for VASARI score and traditional statistics method; in particular, we focused on the AUC value to compare diagnostic accuracy.

The analysis has been done on the software R, using the package ROSE for the balancing purpose.

Applying more or less complex machine learning methods to this type of data is very risky because the more unbalanced the classes, the greater the risk of having results biased by lack of observation in one class.

More specifically, the classification problem’s confusion matrix indicates how well our model classifies the target classes, and it is from this confusion matrix that we derive the model’s accuracy, which is determined by dividing the total number of predictions made by the model correctly by the total number of predictions. Thus, in cases where a class has few observations, it may be categorized as the most popular class, potentially yielding a high accuracy score. For example, one of the most often used parametric techniques for binary classification is logistic regression, which is heavily biased in cases when the classes are not balanced because it underestimates the conditional probabilities of the rare class. To solve these problems, many methods have been proposed in the literature, such as oversampling, undersampling, SMOTE (Synthetic Minority Oversampling Technique), and ROSE (Random Over-Sampling Examples). In this paper, we chose to use the most recent ROSE technique. It is a bootstrap-based method that helps with binary classification when there are uncommon classes present. By creating synthetic examples from a conditional density estimate of the two classes, it can handle both continuous and categorical data. We selected this approach because of the strong theoretical underpinnings of ROSE. It also draws synthetic examples from an estimate of the conditional density underlying the data, thus providing confidence that the distribution of the data into the classes has not changed because the balancement has been performed.

## 3 Results

In previous studies, it has been demonstrated that some of that specific MRI features [enhancement quality (F4), tumor-enhancing proportion (F5), tumor–non-enhancing proportion (F6), and necrosis proportion (F7)] can be used to predict the grade and *IDH* status of gliomas, with important prognostic implications. The standardization and improvement of these data can be used for programming machine learning software (28).

### 3.1 Part 1: preliminary analysis and relationship between GRADE/*levels* and *IDH*

**Figure 1:**
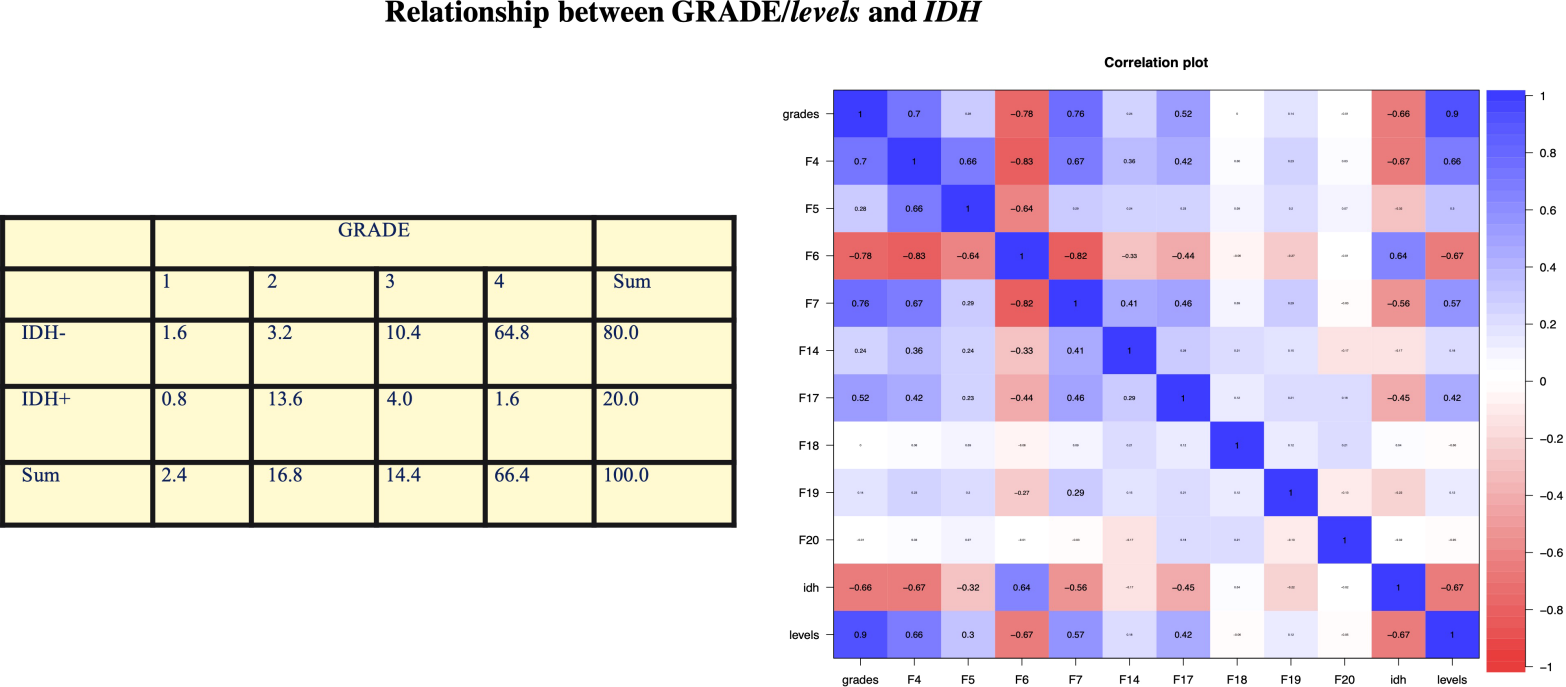
In our analysis, we obtained a clear relationship between negative *IDH* and high GRADE. In fact, 75.2% of the observations have negative *IDH* and high grade (= 3 or 4) with a p-value of 2.2e−16 < 0.05, so the coefficient is statistically significant. We obtained a correlation coefficient of −0.661, which means that these two variables are significantly negatively correlated. The resulting confidence interval is [−0.750, −0.550] at 2.5% and 97.5%, respectively. Statistical analysis by contingency table, which is then confirmed by Pearson’s test, obtained a statistically significant correlation between *IDH* mutation and glioma grade: In 75% of the observations, we found *IDH* WT and high grade.

We obtained a clear relation between a negative *IDH* and high GRADE. In fact, 75.2% of observations have a negative *IDH* and a high grade (= 3 or 4) with a p-value of 2.2e−16 < 0.05, so the coefficient is statistically significant. We obtained a correlation coefficient of −0.661, meaning that these two variables are significantly negatively correlated. The resulting confidence interval is [−0.750, −0.550], respectively, at 2.5% and 97.5%. These results are shown in **Figure 1**.

### 3.2 Part 2: analysis of the statistically significant variables in the prediction of *levels/grade*

We applied this model to the test set to evaluate the predictive performances, and we obtain accuracy = 0.811, sensitivity = 0.781, and specificity = 1, with AUC = 0.906. We proceeded with the analysis of the statistical significance of each variable in the prediction of *levels.* We obtained that variable F4 is significant with a p-value = 2.7e−10 < 0.05 with no significant differences between levels 1 and 3. For every one unit increase in F4 = 2, the odds of being in the level = 1 (high grade) increases by a factor of 4.699.

We calculated the predicted probabilities to be in the high-level grades (vs. low level) at each level of F4: F4 = 3 has 100% probability to be in the high level (grades 3 and 4), whereas the probability for the other two levels is much lower (5% and 20%). This model has accuracy = 0.811, sensitivity = 0.781, and specificity = 1.000, with AUC = 0.869, which is superior to that of the first study (0.73).

Variable F5 is statistically significant with a p-value < 2e−16 < 0.05 with significant differences between levels 2 and 6 of the F5 variable and cutoff level F5 = 4.

For every one unit increase in F5 = 3, the odds of being in the level = 1 (high grade) increases by a factor of 0.49.

F5 = 5 and F6 = 6 have a probability to be in the high level (grades 3 and 4) of 73% and 63%, whereas the probability for the other three levels is much lower (19%, 73%, and 63%).

This model has accuracy = 0.672, sensitivity = 0.908, and specificity = 0.438, with AUC = 0.712.

Variable F6 is statistically significant with a p-value = 0.01409 < 0.05.

For every one unit increase in F6 = 2, the odds of being in the level = 1 (high grade) increases by a factor of 1. We also obtained that there is a significant difference between the F6 = 3 and F6 = 4 (p-value = 0.0049 < 0.05). We calculated the predicted probabilities to be in the high-level grades (vs. low level) at each level of F6: F6 = 1, F6 = 2, and F6 = 3 have a probability to be in the high level (grades 3 and 4) of 100%, whereas the probability for the other levels is much lower (24.1%, 15.9%, and 0%, respectively). We can also clearly identify a cutoff level for this variable. This model has accuracy = 0.757, sensitivity = 0.719, and specificity = 1.000, with AUC = 0.8969. Also, this result is superior to that of our first study.

Variable F7 is statistically significant with a p-value = 2e−16 > 0.05, but none of the levels of F7 is statistically significant. For every one unit increase in F7 = 2, the odds of being in the level = 1 (high grade) increases by a factor of 3.35; whereas for every one unit increase in F7 = 4, the odds of being in the level = 1 (high grade) increases by a factor of 3.22e+09.

Moreover, we calculated the predicted probabilities to be in the high-level grades (vs. low level) at each level of F7: F7 = 4, F7 = 5, and F7 = 6 have the probability to be in the high level (grades 3 and 4) of 100%; whereas when F7 = 1 and F7 = 2, the probability to be in the high level is 8.9% and 24.6%, respectively. This model has accuracy = 0.837, sensitivity = 0.906, and specificity 0.400, with AUC = 0.8 (vs. an AUC of 0.738).

Variable F17 is statistically significant with a p-value = 3.634e−05 < 0.05. Moreover, there is a significant difference between levels F17 = 1 and F17 = 2 with a p-value of 0.017 < 0.05 and also between F17 = 1 and F17 = 3 with a p-value of 0.0017 < 0.05. We can then identify a cufoff in this case.

For every one unit increase in F17 = 2, the odds of being in the level = 1 (high grade) increases by a factor of 7.71; whereas for every one unit increase in F17 = 3, the odds of being in the level = 1 (high grade) increases by a factor of 18.48.

We can calculate the predicted probabilities to be in the high-level grades (vs. low level) at each level of F17: F17 = 3 has the probability to be in the high level (grades 3 and 4) of 74%; whereas when F17 = 1 and F17 = 2, the probability to be in the high level is 13.4% and 24.3%, respectively.

This model has accuracy = 0.840, sensitivity = 0.921, and specificity 0.500, with AUC = 0.8.

**Figure 2:**
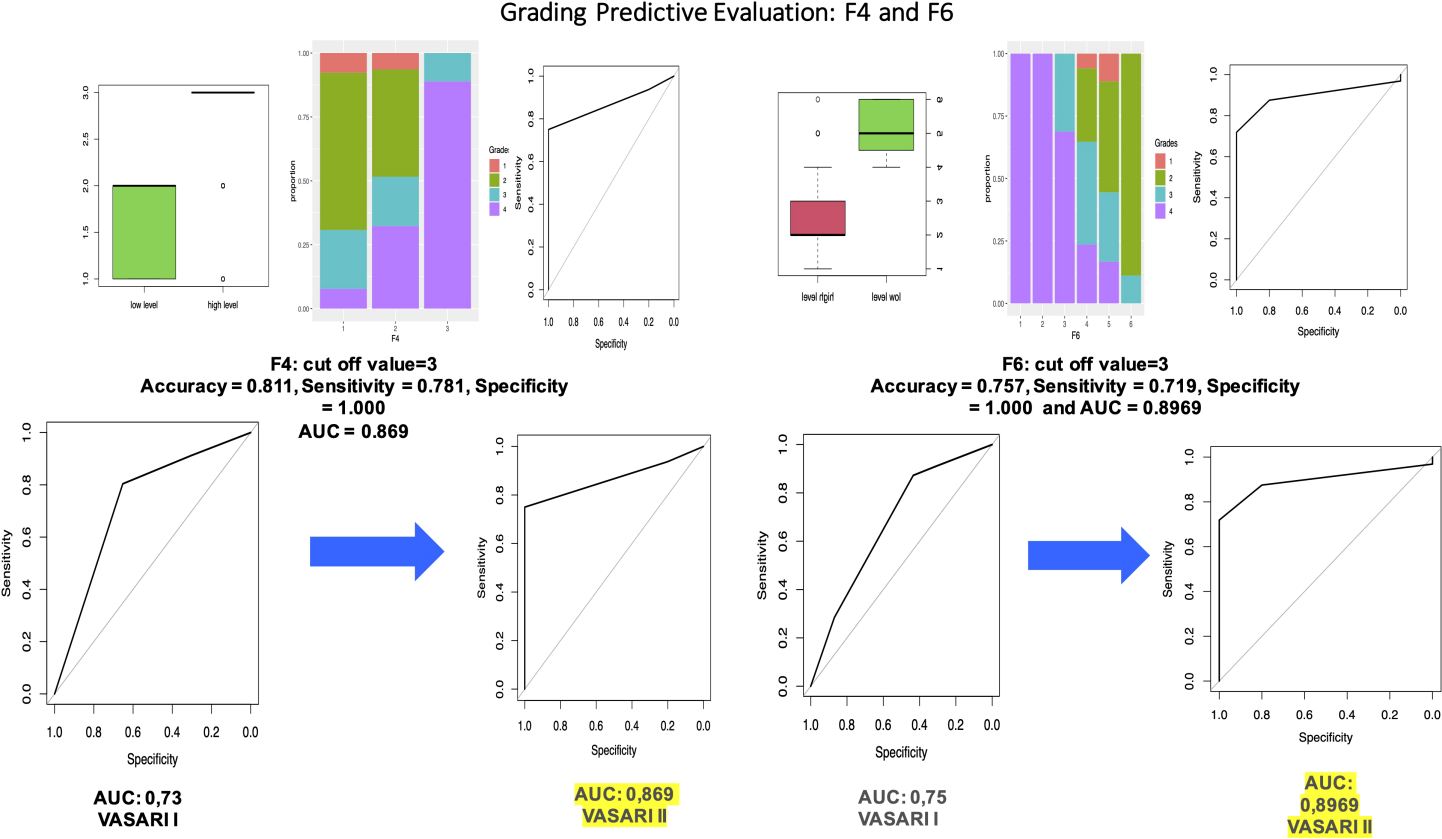
Through logistic regression using the dichotomous variable (high or low grade) as the response variable and F4 as the covariate, we obtained that variable F4 is significant with a p-value = 0.000157 < 0.05. We also compared the performance of VASARI 2.0 with that of traditional VASARI in predicting grade.

Variables F14, F15, F18, F19, and F20 were not statistically significant with a p-value < 0.05. These results are shown in **Figure 2**.

### 3.3 Part 3: analysis of the statistically significant variables in the prediction of *IDH* mutation

The pre-processing part is identical to the one described in part 2: We divided the dataset into train (70%) and test (30%), and, then, we balanced the training data in such a way that we obtained 2,000 observation and perfectly balanced classes (p = 0.5). Then, we conducted a multiple logistic regression and obtained that all the variables are statistically significant. We then applied this model to the test set to evaluate the predictive performances, and we obtained accuracy = 0.88, sensitivity = 0.6, and specificity = 0.95, with AUC = 0.93. We proceed with the analysis of the significance of each variable in the prediction of *IDH*. We obtained that variable F4 is significant with a p-value = 1.9e−05 < 0.05 with a significant difference between F4 = 2 and F4 = 3 (p-value 8.9e−05 < 0.05). The results of the OR have a key role in interpreting the role of the variable. In particular, the OR column means that, for every one unit increase in F4 = 2, the odds of having a positive *IDH* increases by a factor of 0.14; whereas for every one unit increase in F4 = 3, the odds of having a positive *IDH* increases by a factor of 0.0. Moreover, we can calculate the predicted probabilities of having a positive *IDH* (vs. negative) at each level of F4: F4 = 3 has 1.2% probability of having a positive *IDH*, whereas the probability for the other two levels is higher (76.9% and 45.2%). This model has accuracy = 0.90, sensitivity = 0.9, and specificity 0.90, with AUC = 0.904, which is superior to that of our first study (0.73).

Variable F5 is statistically significant with a p-value = 0.04386 < 0.05 with a significant difference also between F5 = 3 and F5 = 4 with a p-value = 3.6e−11 < 0.05 and between F5 = 3 and F5 = 5 with a p-value = 0.0077 < 0.05. Hence, we can conclude that the cutoff level for variable F5 is F5 = 3. The OR column showed that, for every one unit increase in F5 = 2, the odds of having a positive *IDH* increases by a factor of 4.978539e−08; whereas for every one unit increase in F5 = 7, the odds of having a positive *IDH* increases by a factor of 5.524675e−16. These values are so low because F5 = 1 is taken as baseline, where only positive values are observed.

Moreover, we calculated the predicted probabilities of having a positive *IDH* (vs. negative) at each level of F5: F5 = 1, F5 = 2, and F5 = 3 have a high probability to have a positive *IDH* of 100%, 67%, and 81%, respectively, whereas the probability for the other three levels is much lower (53.3%, 14%, and ~0%). This modes has accuracy = 0.808, sensitivity = 0.48, and specificity 0.890, with AUC = 0.73.

Variable F6 is statistically significant with a p-value = 5.162e−0 5< 0.05 with a significant difference between levels F3 and F4 with a p-value of 2e−16 < 0.05 and between F5 and F6 with a p-value of 1.1e−14 < 0.05. With these results, we may identify two different cutoffs.

The OR column showed that, for every one unit increase in F6 = 2, the odds of having a positive *IDH* increases by a factor of 1; whereas for every one unit increase in F6 = 4, the odds of having a positive *IDH* increases by a of 6.704338e+08.

Moreover, we calculated the predicted probabilities to be in the high-level grades (vs. low level) at each level of F6: F6 = 4, F6 = 5, and F6 = 6 have the highest probability to have a positive *IDH*: 68.1%, 75.5%, and 97.4%, respectively, whereas the probability for the other levels is 0%. This model has accuracy = 0.86, sensitivity = 0.90, and specificity = 0.85, with AUC = 0.9125 (vs. AUC = 0.7648).

We obtained that variable F7 is statistically significant with a p-value = 2.31e−06 < 0.05 and a significant difference between F7 = 3 and F4 = 4 (p-value = 0.00085 < 0.05). Another significant difference is between F7 = 2 and F7 = 4. We can then conclude that we have a cutoff for values higher than F7 = 3. The OR column showed that, for every one unit increase in F7 = 2, the odds having a positive *IDH* increases by a factor of 2.083333e−01. Moreover, we can calculate the predicted probabilities to have a positive *IDH* at each level of F7: F7 = 1, F7 = 2, and F7 = 3 have a probability of having a positive *IDH* of 61.5%, 25%, and 21.2%, respectively, whereas F7 = 4, F7 = 5, and F7 = 6 have a probability of having a positive *IDH* mush lower: 0%. This model has accuracy = 0.740, sensitivity = 1.000, and specificity = 0.675, with AUC = 0.835 (vs. AUC = 0.789).

Variable F14 is barely statistically significant with a p-value = 0.0648 > 0.05 with no significant difference between the levels in F14. The OR column showed that, for every one unit increase in F14 = 2, the odds having a positive *IDH* increases by a factor of 0.25; whereas for every one unit increase in F14 = 3, the odds of being in the odds having a positive *IDH* increases by a factor of 0.625. Moreover, we can calculate the predicted probabilities to have a positive *IDH* at each level of F14: F14 = 3 has the highest probability of having a positive *IDH* with the 38.5%; whereas F14 = 2, F14 = 4, and F14 = 5 have a probability of having a positive *IDH* much lower: 20%, 8.8%, and 12.5%, respectively. This model has accuracy = 0.780, sensitivity = 0.600, and specificity 0.825, with AUC = 0.75.

Variable F17 is statistically significant with a p-value = 9.372e−06 < 0.05 and a significant difference between the levels in F17 = 1 and F17 = 2 with a p-value of 0.00049 < 0.05. The OR column showed that, for every one unit increase in F17 = 2, the odds having a positive *IDH* increases by a factor of 0.11; whereas for every one unit increase in F17 = 3, the odds of being in the odds having a positive *IDH* increases by a factor of 0.05. Moreover, we can calculate the predicted probabilities to have a positive *IDH* at each level of F17: F17 = 3 has the lowest probability of having a positive *IDH* with the 8.7%; whereas F17 = 1 has a higher probability of having positive *IDH*: 65%. This modes has accuracy = 0.860, sensitivity = 0.400, and specificity 0.975, with AUC = 0.7925.

**Figure 3:**
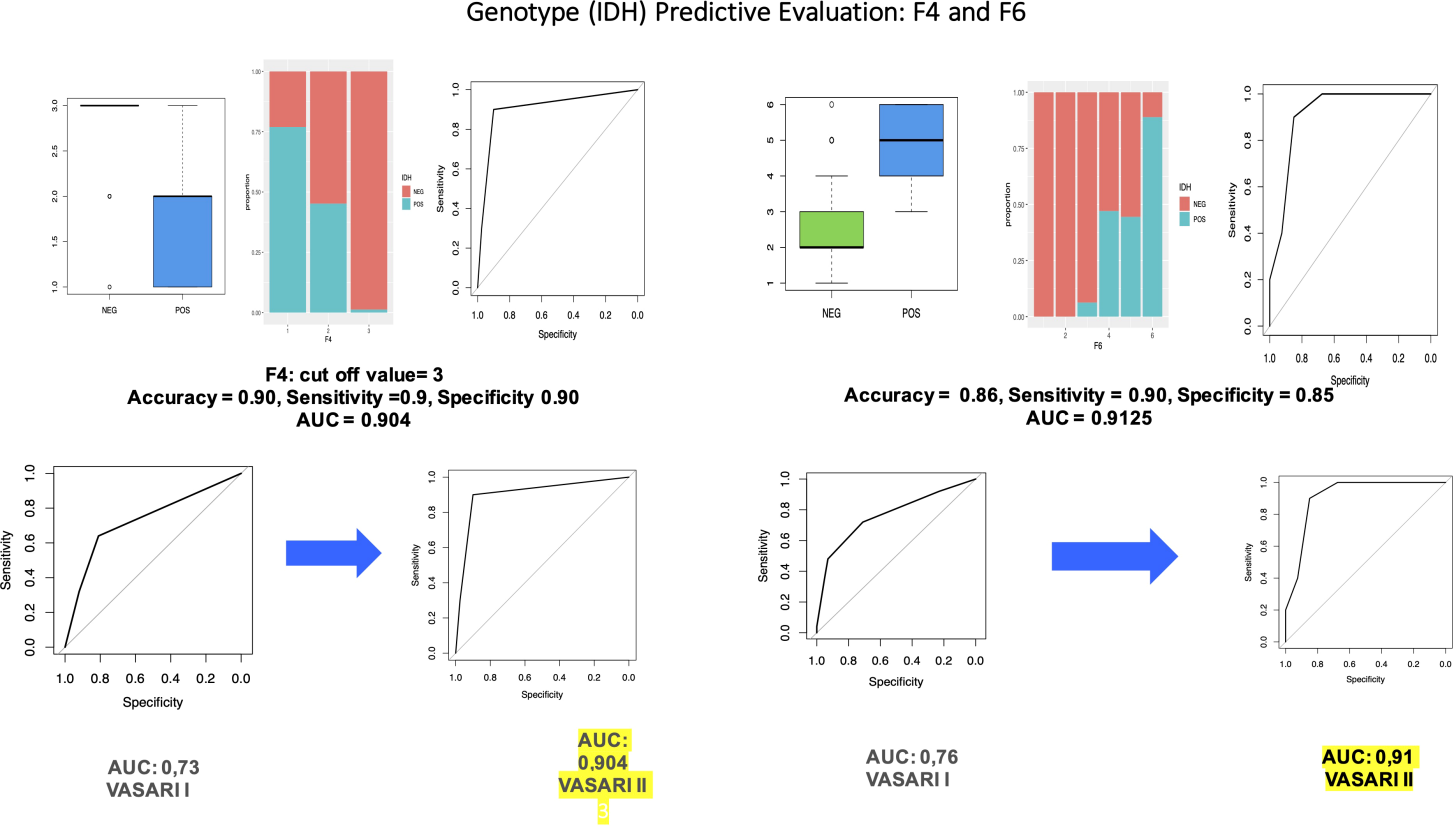
Through univariate logistic regression to evaluate the influence of variables in predicting/*IDH* levels, we obtained that variable F6 is statistically significant with a p-value = 0.0032 < 0.05. We also performed a Wald test on variable F6, which concluded that this variable is statistically significant (p-value = 0.01409 < 0.05). We also compared the performance of VASARI 2.0 with that of traditional VASARI in predicting *IDH* status.

Other features, qualitative or non-statistically significant, were not taken into account. These results are shown in **Figure 3**.

## 4 Discussion

In our study, we confirm that there is a positive statistical evidence between some VASARI features and *IDH* and glioma grade (**Figure 1**). The most significant variables in the prediction of *IDH* are F4 (AUC: 0.904), F5 (AUC: 0.73), F6 (AUC: 0.91), F7 (AUC: 0.84), F14 (AUC: 0.75), and F17 (AUC: 0.79).

The most significant variables in the prediction of *levels* are F4 (AUC: 0.87), F5 (AUC: 0.7), F6 (AUC: 0.89), F7 (AUC: 0.79), and F17 (AUC: 0.75).

The statistical significance for all the features is increased using VASARI 2.0 compared to result obtained with traditional VASARI ranges, demonstrating how the new proposed VASARI lexicon promotes an increase in the sensitivity, specificity, and AUC of these features to increase the statistical significance with AUC > 0.8 and to make this system more suitable for clinical practice. We can distinguish low-grade gliomas from high-grade gliomas using this model. Numerous studies have assessed the application of the VASARI Lexicon in the categorization of cerebral gliomas (29–34). Due to a much more extensive destruction of the blood–brain barrier in high-grade gliomas compared to that in low-grade gliomas, our study, for instance, produced similar results to those in the work done by Su et al., in which the enhancement quality and the proportion enhancing were significantly higher in high-grade gliomas (33). High-grade gliomas exhibit much higher cell growth proliferation. In the context of tumor tissue, new, irregular, and aberrant vessels form very rapidly, without an adequate blood–brain barrier, a process known as neoangiogenesis. Newly formed vessels are much more permeable than normal vessels. Therefore, in an MRI study after intra venous of contrast agent, the solid tumor component appears much brighter or “enhanced” in the resulting images, given that the contrast agent can cross the blood–brain barrier very easily, escaping from the blood vessels into the surrounding tumor tissue. Furthermore, areas of necrosis can very often be found in high-grade gliomas, given that they have a very high rate of proliferation and cell growth, which is associated with an inadequate blood supply. These necrotic phenomena, therefore, contribute to the breakdown of the blood–brain barrier, allowing a greater leakage of contrast agents into the tumor tissue and promoting greater further enhancement (31, 32). In high-grade gliomas, there are often infiltrative lesional margins in the surrounding healthy tissue. In MRI, all this appears as an area of pathological alteration of the signal intensity, with hyperintensity in the Fluid Attenuated Inversion Recovery (FLAIR)-weighted sequences, much larger and more extensive than the pathological area visible on the T1-weighted MRI sequences. Our study supports this theory by showing that the infiltrative T1/FLAIR ratio has a high predictive value for glioma grade (OR = 41.99, p = 0.001). The fast and uncontrolled phenomenon of neoangiogenesis is indirectly indicated by the presence of hemorrhagic components and necrosis in the context of high-grade gliomas. The newly created blood vessels are easily injured and do not have an uneven morphology, which results in bleeding. In our study, the presence of necrosis was a strong predictive factor with an OR of 13.57 (p < 0.001) for high-grade glioma, a result that is consistent with previous research findings (30–34).

In our study, we found that edema proportion was a significant factor. These findings, in line with other recent studies, highlight that mass effect is an important predictor of astrocytoma grade. Tumor perilesional cerebral edema correlates with the WHO pathological grading as recently demonstrated (35). Measuring areas of non-enhancing tumor have been highlighted by The Response Assessment in Neuro-Oncology Working Group (36). Astrocytomas represent very heterogeneous neoplasms. The component characterized by contrast enhancement does not always contain anaplastic parts, unlike the component without contrast enhancement, which often contains both anaplastic parts and low-grade parts. The evaluation of the nCET component, therefore, is very important both in the diagnosis and in the follow-up to better assess the therapeutic monitoring of astrocytoma. However, understanding the real extent of a high-grade astrocytoma by evaluating the nCET proportion is very difficult given the extremely heterogeneous nature of the tumor and the extreme difficulty in delineating its peripheral margins. In reality, all this represents a false dichotomy, given that, in infiltrating gliomas, there is an “infiltrative edema,” consisting of tumor cells and edema in the background of the brain. Furthermore, even with the use of sophisticated techniques like T2 mapping, diffusion tensor imaging, and perfusion imaging, it remains difficult to differentiate pure vasogenic edema from infiltrative edema. In this work, using multivariate analysis based on VASARI, we showed that nCET percentage was a predictive factor of grade 4 astrocytoma. The new classification of tumors of the central nervous system published in 2021 highlights the importance of evaluating the molecular status, particularly, and first of all, the *IDH* mutation. Usually, one of the two *IDH* genes, *IDH1* and *IDH2*, is affected by the mutation. The mutation most frequently found in gliomas is that affecting the *IDH*1 gene. Typically, a specific mutation (R123H) occurs that causes a single–amino acid change in the active enzyme site. The mutated *IDH* enzyme promotes the conversion of alpha ketoglutarate into 2-hydroxyglutarate, an oncological metabolite that induces cancer formation (6). The presence of the *IDH* mutation is associated with a significantly better prognosis compared to *IDH*–wild-type gliomas, and its identification is, therefore, very important for the classification of cerebral gliomas and for clinical therapeutic management. In our study, six VASARI features were found to predict *IDH* mutation status: F4, enhancement quality (AUC: 0.904); F5, tumor-enhancing proportion (AUC: 0.73); F6, tumor–non-enhancing proportion (AUC: 0.91); F7, necrosis proportion (AUC: 0.84); F14, proportion of edema (AUC: 0.75); and F17, diffusion characteristics (AUC: 0.79). Our study confirms the results of our recently published study for VASARI characteristics and demonstrates an increase in their diagnostic accuracy, especially regarding F4 and F6 with an AUC value greater than 85%, in line with the ESR statement on the validation of imaging biomarkers (European Society of Radiology, 2020). Olar et al. identified a significant correlation between proportion of enhancing tumor and *IDH* mutation. In the study, the researchers studied the role that the *IDH* mutation may have in the grading and mitotic index in grade II–III diffuse astrocytomas, and their results demonstrated that the *IDH* mutation determines the effect of mitotic index on patient outcome (37, 38). *IDH*–wild-type gliomas usually have a higher contrast enhancement than *IDH*-mutant gliomas (39–43), and *IDH* wild-type gliomas have undefined margins (44). Weighted FLAIR sequences are very useful for evaluating areas with pathological signal intensities. In the case of *IDH*–wild-type gliomas, the areas with pathological signal hyperintensity in the weighted FLAIR sequences, which extend beyond the margins of enhancement, usually represent the infiltrative edema component, characterized by the presence of infiltration of tumor cells in the peripheral tissue (31). Using multivariate analysis, we discovered that, in our cohort, the percentage of necrosis accurately predicts the status of *IDH* mutation. In our investigation, necrosis accounted for less than 25% of the total tumor volume in *IDH*-mutant cohorts and more than 50% in *IDH*-wild phenotypes. These results support the conclusions of multiple investigations. *IDH*-mutants were frequently linked to a cutoff necrosis of less than 33% of the tumor, according to Park et al. (45). Excessive tumor necrosis in *IDH*–wild-type gliomas is determined by increased hypoxia, which is brought on by intravascular thrombosis and the coagulation pathway activation (45–47). Furthermore, our results highlight two other VASARI features that can be used in the prediction of *IDH* status, which were not highlighted in our recently published work, namely, F14 (proportion of edema) and F17 (diffusion characteristics). In *IDH*-mutant gliomas, absent edema or edema with an extension smaller than that of the solid tumor component was found; whereas in *IDH*–wild-type gliomas, the extension of the edematous alteration was greater than or equal to the volume of the tumor. Similar results were documented in the studies by Lasocki et al. (40) and Patel et al. (48). Lasocki et al. found a cutoff value of 33% to distinguish *IDH*-mutant gliomas from *IDH*–wild-type gliomas. Furthermore, the presence of cysts was documented more frequently in *IDH*-mutant gliomas, in line with other published studies (49). In several other studies, *IDH*-mutant gliomas had higher average ADC values ​​than *IDH*–wild-type gliomas, underlining that their edematous component is usually less infiltrative and destructive (50–54). Nowadays, the main attraction of the scientific interest is radiomic and machine learning that has been applied to tumor grading and diagnosis, tumor segmentation, non-invasive genomic biomarker identification, detection of progression, and patient survival prediction. It has been suggested that machine learning models are capable of more accurate prognosis prediction than histopathological categorization. These considerations could be the starting point for subsequent studies. The standardization and improvement of these data can be used for programming machine learning software (55). In the field of brain tumor, interest in machine learning methods is increasing, especially in diagnosis and pre-surgery planning though un-invasive histopathological categorization. Radiomics may be able to determine a tumor’s response to treatment, make an accurate diagnosis, and forecast a prognosis (56). In addition to offering extra prognostic data, radiomic analysis’ ability to non-invasively differentiate between various glioma molecular subtypes would aid in the choice of targeted chemotherapy for patients with numerous genetic mutations and possibly high-grade tumor types (57–59). Additionally, it would improve surgery, which is necessary to maintain median survival (60). Therefore, treatment responses, progression-free survival, and overall survival can all be more accurately predicted with the application of radiomic risk models (61, 62). It is possible to evaluate the effectiveness of anti-angiogenic therapy without endangering the patient by non-invasively acquiring the radio-genomic profile of a tumor (63). Our study has some limitations that need to be clarified and discussed: a single retrospective center study and a small sample size. This would not allow a validation of the new VASARI 2.0 method proposed by use on a large scale quickly. However, the number of cases is in line with for the type of analysis described. Future studies with multi-center data or larger cohorts needed for a full validation of the new VASARI lexicon that we proposes, in order to eliminate the risk of data bias, which could affect the generalizability of the study results. However, there are also strong points for the use of the proposed VASARI 2.0 lexicon in daily clinical radiological practice. It does not require specific software to automate the scoring process. There are methodological innovations in the evaluation of the MRI VASARI features, thanks to adequate changes in the reference intervals as reported in **Table 1**. Familiarization with this new lexicon is easy. It can be easily used in daily clinical practice also because it can be of valid help in capturing the most salient aspects to be described in the report. It can represent a valuable tool for producing a structured and standardized report with the aim of offering with simplicity and clarity all the salient information needed by the neuro-oncology core group (oncologist, radiotherapist, neurosurgeon, and neuoradiologist). It would be desirable to conduct a large-scale multi-center study to then draft a new VASARI lexicon guide based on the validation results.

## 5 Conclusion

The evaluation of gliomas with modified ranges/score of VASARI 2.0 allows the prediction of the outpoint (*IDH* status and grade) with AUC > 0.8, higher than that of traditional VASARI. Thus, the new score could be used in pre-surgical evaluation of gliomas in a method both suitable with clinical practice and that can also be the starting point for subsequent studies of radiomics and machine learning.

## Data Availability

All relevant data is contained within the article. Additional data are available upon request to interested researchers.
